# Branding Asklepios and the Traditional and Variant Serpent Symbol Display Among Health Professional Schools in the United States, Puerto Rico, and Canada: A Cross-Sectional Survey

**DOI:** 10.2196/mededu.5515

**Published:** 2016-05-25

**Authors:** Claus Hamann, MaryKate Martelon

**Affiliations:** ^1^ Eastern Maine Healthcare Systems Beacon Health Brewer, ME United States; ^2^ Massachusetts Department of Public Health Boston, MA United States

**Keywords:** caduceus, emblems, insigne, insignia, history of medicine, history, humanities, Asklepios, Asclepius, medical symbol

## Abstract

**Background:**

History supports the staff and single serpent, the asklepian, as the symbol of healing and medicine, yet its confusion with the caduceus (a winged staff with two snakes wrapped around it) persists. No population-based information on serpent symbol use exists.

**Objective:**

To determine the prevalence of asklepian and caduceus display among Internet images of medical and health professional schools’ emblems, and to compare asklepian and caduceus display between medical and health professional schools, examining the effects of school longevity and geographic location on symbol display.

**Methods:**

This cross-sectional survey examined Internet websites and Google Images associated with medical and other health professional schools in the United States, Puerto Rico, and Canada from 2013 to 2015. The primary outcome was display of a traditional or variant asklepian or caduceus among current and past emblems in Google Images. Odds ratios (ORs) and 95% confidence intervals for the comparison of medical versus other health professional schools were calculated by logistic regression. Differences among schools' longevity were assessed with Student's *t*-tests and linear regression.

**Results:**

Among images of current and past emblems of 482 schools—159 medical schools and 323 health professional schools—107 (22.2%) emblems displayed only the traditional, and 205 (42.5%) any, asklepian. Adjusting for geographic region and longevity, medical schools were 59% less likely than health professional schools to display the traditional asklepian (OR 0.41, 95% CI 0.24-0.71, *P*=.001), and were 7.7 times more likely than health professional schools to display the traditional caduceus. Medical schools were 8% less likely than health professional schools to display any asklepian (OR 0.92, 95% CI 0.62-1.38, *P*=.70), and were 3.3 times more likely than health professional schools to display any caduceus.

**Conclusions:**

Schools’ preference of the asklepian over the caduceus confirmed historical origins. Less asklepian and more caduceus display by medical schools suggests an educational opportunity for the medical profession to define for itself and the public the correct symbol of an interdisciplinary mission of healing.

## Introduction

**Figure 1:**
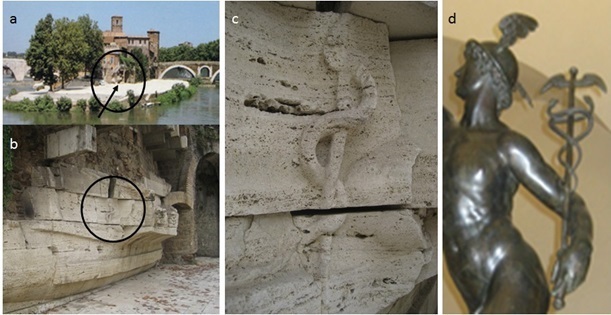
Asklepian from a Roman Aesculapian temple and caduceus from a Florentine sculpture. (a) Tiber Island, Rome 2004 [2]. The arrow indicates the travertine ship's prow, which is seen in (b,c); (b,c) Asklepian carved into the travertine ship's prow from the 1st century BCE at the site of an Aesculapian temple [2], 300 m from the current-day Ospedale Fatebenefratelli (photo by author, 2008); (d) Bronze by Gianbologna, Mercurio (Mercury, the Roman name for Hermes), 1580, Museo Nazionale del Bargello, Florence, Italy (excerpt of photo by author, 2013).

For more than two millennia, the single serpent of Epidauros and staff of Asklepios—a combination named the asklepian [1]—have symbolized healing and medicine in the Greco-Roman tradition (see Figure 1, a-c, for photos of these symbols [2]). For two centuries, the Sumerian-derived caduceus of Hermes (ie, Mercury) [3], the messenger god (a winged staff with two snakes wrapped around it, see Figure 1, d), has been displayed by many health-related organizations as a quasi-symbol of health care since its use by a nineteenth-century medical publisher [1]. This symbol substitution was accelerated by adoption of the caduceus as an insigne for noncombatant officers of the US Army Medical Corps in 1902 [4] despite the Corps' use of the asklepian since 1818 [5]. *Le caducée* has been used to describe the single serpent entwining fascicles [6] and the asklepian has been misnamed “the medical caduceus” [1,7]. The US error, explained in 1917 [5], has been discussed in at least 30 articles for a century [8]. Confusion as to the correct symbol for healing, medicine, and health care persists in both professional and popular usage.

The distinctive meanings of these mythological symbols are well described. The traditional, pruned branch-like staff of Asklepios and its single entwined serpent each represent healing and restoration through regeneration: new twigs growing from a pruned branch and the snake shedding its former skin [9]. Hippocrates was known as an Asclepiad [10] and graduands invoke Asklepios in the traditional Hippocratic oath [11]. The caduceus of Hermes, a smooth, winged, herald’s wand with two entwined serpents, is associated with communication, wisdom, peace, commerce, alchemy, thievery, and tangentially with healing [6].

Medical and kindred health professional schools—where initial exposure to these symbols occurs—are among the “professional medical organizations...more likely to use the staff of Aesculapius” [6], though selection criteria, analytic method, and calculations were not described by the author. A detailed pictorial history of the asklepian [12] and a survey [13] provided only qualitative support for preference of the asklepian over the caduceus by medical and health organizations.

To the authors’ knowledge, only one other study has examined the display of these symbols. Among the 10 leading medical colleges in India, 1 displayed the asklepian, 6 used the caduceus, and 3 used neither [14]. To inform current and future use of these symbols by medical and health professional schools, other health care organizations, and the public, this study asks the following questions: Do schools display the asklepian more than the caduceus? Do medical schools display either symbol more than do health professional schools? Do school longevity or geographic location influence display of either symbol?

**Figure 2:**
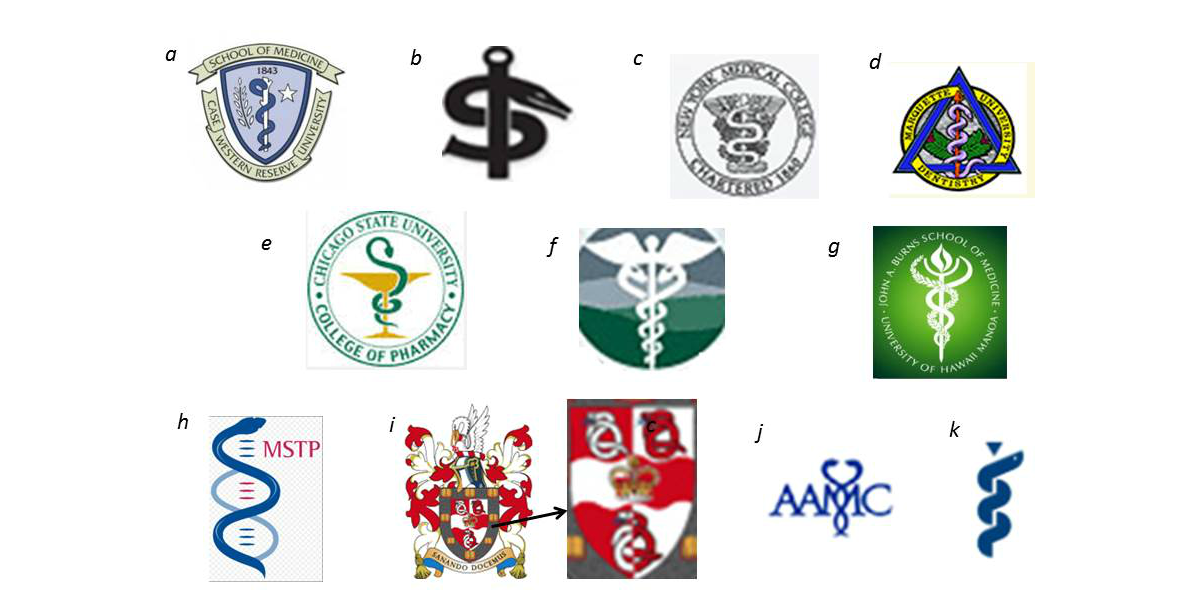
a [28], d [31], e [32] traditional asklepians; b [29], c [30], i [36] variant asklepians; f [33] traditional caduceus; g [34] variant caduceus; h [35] double-helix in emblem; j [37], k [38] change in AAMC emblem.

## Methods

From November 2013 to January 2015, we compared Internet displays of asklepians and caducei among emblems of all accredited American, Puerto Rican, and Canadian allopathic medical schools [15] to those of all accredited schools of osteopathic, podiatric, and veterinary medicine, and of dentistry, optometry, and pharmacy [16-25]. The primary outcome was the display of a traditional, or any variant of a traditional, asklepian or caduceus among Google Images [26] of current or past emblems associated with a school, its departments, or organizations. We also examined the display of symbols in each school’s current emblem on its home page.

The primary author (CH) searched Google Images, which ranks images according to keywords, richness of text descriptions, and website links for each image [26]. Images were searched up to the “Show more results” line at the bottom of the webpage. This strategy included, on average, the first 398 images (SD 3.3) based on a 1% random sample. Each home page of all medical and health professional schools in the respective directories was also accessed via the link provided in the directory or via the school's name entered in the Google Web search field. To obtain images (ie, screenshots) of emblems, each name was entered in the Google Images search field. Sites displaying either symbol were again accessed between November 2014 and January 2015 to verify active display; Google Image search [27] was conducted for inactive Web addresses. For all schools, the same emblems displayed on home pages were also found in searches for images.

Examples of traditional and variant asklepians and caducei are shown in Figure 2, a-k [28-38]. We defined the traditional asklepian as the display of the branch-like staff and a single serpent (see Figure 2, a), regardless of serpent chirality, number of coils, or ornamentation. Dentistry’s emblem is the dental cautery—equivalent of the asklepian staff [39]—with entwined single serpent. Additional features of dentistry’s emblem are the berries and leaves that represent temporary and permanent teeth, respectively, and the Greek letters omicron, odont (ie, tooth), and delta (ie, dentistry) (see Figure 2, d). For pharmacy, the asklepian equivalent was defined as a display of the bowl of Hygieia, a daughter of Asklepios [40], with an entwined single serpent [41] (see Figure 2, e). We defined the traditional caduceus as two mirror-image serpents entwining a smooth, winged wand (see Figure 2, f), regardless of the number of coils or ornamentation. In addition to the traditional asklepian and caduceus, we designated two asklepian variants (see Figure 2, b, c, and i) and one caduceus variant (see Figure 2, g and j), according to staff and wand features described in Multimedia Appendix 1. Author agreement on traditional and variant symbols was reliable at a kappa [42] of .91.

We defined each medical and health professional school's major US census region [43] as follows: Puerto Rican schools were grouped into the South region; British Columbia and Alberta were grouped into the West region; Saskatchewan and Manitoba were grouped into the Midwest region; and the remaining Canadian provinces were grouped into the Northeast region. School longevity was defined as the founding year subtracted from 2014. Each school’s founding year was identified from its home page under the *About Us/History* tab or by entering the school’s name and the words “founded in” in the Google Web search field.

Current and past emblems from images and current emblems from home pages were analyzed independently. Odds ratios (ORs) (95% CI) for asklepian versus caduceus display and for interaction effects of geographic region and longevity on the relationship between school type and symbol display were calculated by logistic regression. School longevity was normally distributed and calculated as mean (SD). Differences among schools' longevity were assessed with Student's *t*-tests and linear regression. Statistical significance was asserted at <.05; all statistical tests were two-tailed. Analyses were performed with Stata version 13.1 (StataCorp LP, College Station, TX). The institutional review board of Mercy Medical Center, Springfield, MA, waived review of this study.

## Results

Among images of current and past emblems of 482 schools—159 medical schools (33.0%) and 323 health professional schools (67.0%)—107 (22.2%) displayed traditional asklepians and 205 (42.5%) displayed any asklepian (see Table 1). A total of 18 of the 482 schools (3.7%) displayed the traditional caduceus; 25 (5.2%) displayed any caduceus. A total of 249 schools (51.7%) displayed neither symbol.

Adjusting for geographic region and longevity, medical schools were 59% less likely than health professional schools to display traditional asklepians (OR 0.41, 95% CI 0.24-0.71, *P*=.001) (see Table 2, *Current and past emblems in Google Images*), yet were 7.7 times more likely than health professional schools to display the traditional caduceus (95% CI 2.50-23.80, *P*<.001). Medical schools were 8% less likely than health professional schools to display any asklepian (OR 0.92, 95% CI 0.62-1.38, *P*=.70), yet were 3.3 times more likely than health professional schools to display any caduceus (95% CI 1.43-7.75, *P*=.005).

In a secondary analysis of home pages, 36 current emblems of all 482 schools (7.5%) displayed traditional asklepians, and 77 (16.0%) displayed any asklepian (see Table 3). A total of 7 of the 482 schools (1.5%) displayed the traditional caduceus and 8 (1.7%) displayed any caduceus.

Adjusting for geographic region and longevity, medical schools were 62% less likely than health professional schools to display traditional asklepians in current home page emblems—a statistically nonsignificant result (adjusted OR 0.38, 95% CI 0.14-1.02, *P*=.06; see Table 2). The higher odds of caduceus display by medical schools were also not significant.

For all schools in the United States or Puerto Rico compared to Canada, there were no significant differences in the display of asklepian versus caduceus (data not shown). Multimedia Appendices 2 and 3 contain the hyperlinked emblems of all schools displaying the asklepian, caduceus, and variants.

**Table 1:** Display of asklepians and caducei among current and past emblems in Google Images for US and Canadian medical and other health professional schools in 2014.

Schools		Longevity^a^, mean (SD, range)	Traditional asklepian, n (%)	Traditional or variant asklepian, n (%)	Traditional caduceus, n (%)	Traditional or variant caduceus, n (%)	Neither/ both, n (%)
All schools (n=482)		75.8 (58.5, 1-249)	107(22.2)	205 (42.5)	18 (3.7)	25 (5.2)	252 (52.3)
**Medical schools**							
	All (n=159)	98.5(61.3, 1-249)	21 (13.2)	65 (40.9)	14 (8.8)	16(10.1)	78 (49.1)^b^
	US^c^(n=137), PR^d^(n=4)	97.7(62.0, 1-249)	18 (12.8)	54 (38.3)	14 (9.9)	16 (11.3)	71 (50.4)
	Canada (n=18)	104.5 (58.1, 9-185)	3 (17)	11 (61)	0 (0)	0 (0)	7 (39)
**Medical schools: region**							
	Northeast (n=44)	119.9 (72.8, 1-249)	6 (14)	20 (45)	3 (7)	3 (7)	21 (48)
	Midwest (n=33)	97.9 (53.2, 1-178)	4 (12)	14 (42)	2 (6)	2 (6)	17 (52)
	South (n=56)	87.9 (58.8, 2-207)	8 (14)	22 (39)	7 (13)	7 (13)	27 (48)
	West (n=26)	86.0 (47.0, 6-195)	3 (12)	9 (35)	2 (8)	4 (15)	13 (50)
**Other health professional schools: all types**							
	All (n=323)	65.8 (53.5, 1-193)	86(26.6)	140(43.3)	4 (1.2)	9 (2.8)	174 (53.9)^e^
	US (n=287), PR (n=3)	65.1 (53.8, 1-193)	81 (27.9)	130 (44.8)	3(1.0)	7 (2.4)	153 (52.8)
	Canada (n=33)	71.8 (44.0, 5-154)	5 (15)	10 (30)	1 (3)	2 (6)	21 (64)
**Other health professional schools: region**							
	Northeast (n=78)	74.3 (55.3, 1-193)	10 (13)	19 (24)	1 (1)	1 (1)	58 (74)
	Midwest (n=75)	86.9(49.3, 1-164)	19 (25)	33 (44)	1 (1)	4 (5)	38 (51)
	South (n=106)	51.7 (46.7, 1-173)	36 (34.0)	54 (50.9)	0 (0)	1 (0.9)	51 (48.1)
	West (n=64)	53.9 (46.1, 1-144)	21 (33)	34 (53)	2 (3)	3 (5)	27 (42)
**Other health professional schools: osteopathic medicine**							
	All (n=40)	32.3 (33.6, 1-122)	16 (40)	25 (63)	1 (3)	1 (3)	14 (35)
	US (n=34)	33.1 (36.2, 1-122)	16 (47)	25 (74)	0 (0)	0 (0)	9 (26)
	Canada (n=6)	27.5 (11.7, 11-33)	0 (0)	0 (0)	1 (17)	1 (17)	5 (83)
**Other health professional schools: veterinary medicine**							
	All (n=35)	82.5 (45.5, 16-162)	19 (54)	28 (80)	0 (0)	0 (0)	7 (20)
	US (n=30)	82.8 (45.0, 16-162)	17 (57)	24 (80)	0 (0)	0 (0)	6 (20)
	Canada (n=5)	81.0 (58.3, 28-152)	2 (40)	4 (80)	0 (0)	0 (0)	1 (20)
**Other health professional schools: podiatric medicine**							
	All (n=10)	60.9 (47.2, 5-119)	2 (20)	6 (60)	0 (0)	0 (0)	4 (40)
	US (n=9)	66.6 (47.1, 5-119)	2 (22)	6 (67)	0 (0)	0 (0)	3 (33)
	Canada (n=1)	10.0 (N/A^f^)	0 (0)	0 (0)	0 (0)	0 (0)	1 (100)
**Other health professional schools: dentistry**							
	All (n=74)	80.7 (47.9, 1-174)	18 (24)	33 (45)	1 (1)	2 (2)	39 (53)
	US (n=64), PR (n=1)	80.2 (49.7, 1-174)	18 (28)	30 (46)	1 (2)	1 (2)	34 (52)
	Canada (n=9)	83.8 (40.5, 43-139)	0 (0)	3 (33)	0 (0)	1 (11)	5 (56)
**Other health professional schools: pharmacy**							
	All (n=141)	64.0 (55.5, 1-193)	29 (20.6)	44 (31.2)	2 (1.4)	4 (2.8)	93 (66.0)
	US (n=130), PR (n=1)	62.2 (55.8, 1-193)	26 (19.8)	41 (31.3)	2 (1.5)	4 (3.1)	86 (65.6)
	Canada (n=10)	88.6 (49.0, 5-154)	3 (30)	3 (30)	0 (0)	0 (0)	7 (70)
**Other health professional schools: optometry**							
	All (n=23)	65.4 (46.5, 1-168)	2 (9)	4 (17)	0 (0)	2 (9)	17 (74)
	US (n=20), PR (n=1)	64.5 (47.7, 1-168)	2 (10)	4 (19)	0 (0)	2 (10)	15 (71)
	Canada (n=2)	75.5 (51.1, 47-104)	0 (0)	0 (0)	0 (0)	0 (0)	2 (100)

^a^Number of years since founding: *t* =-6.21; *P*<.001.

^b^Includes 3 medical schools (1.9%: the United States) displaying both symbols.

^c^US: the United States.

^d^PR: Puerto Rico.

^e^Includes 2 health professional schools (0.6%: dentistry and pharmacy, both in the United States) displaying both symbols.

^f^N/A: not applicable.

**Table 2:** Adjusted analysis of asklepian and caduceus in the emblems of US, Puerto Rican, and Canadian medical versus other health professional schools in 2014.

Symbols		Traditional asklepian	Traditional or variant (any) asklepian	Traditional caduceus	Traditional or variant (any) caduceus
**Current and past emblems in Google Images**					
	Total, n	107	205	18	25
	Adjusted OR^a,b^	0.41	0.92	7.70	3.32
	95% CI	0.24-0.71	0.62-1.38	2.50-23.80	1.43-7.75
	*P*	.001	.70	<.001	.005
**Current emblems on home pages**					
	Total, n	36^c^	77^d^	7	8
	Adjusted OR^b^	0.38	1.08	2.75	1.92
	95% CI	0.14-1.02	0.62-1.89	0.61-12.50	0.47-7.80
	*P*	.06	.79	.19	.36

^a^OR: odds ratio.

^b^Adjusted for school's geographic region and longevity; health professional school was the reference variable. We found no significant interaction effects in the analysis of current and past emblems in Google Images.

^c^Significant interaction effects in the analysis of current emblems on home pages were newer health professional schools displaying traditional asklepian more than older health professional schools (OR 1.020, 95% CI 1.002-1.040, *P*=.03).

^d^Significant interaction effects in the analysis of current emblems on home pages were western medical schools displaying any asklepian less than western health professional schools (OR 0.22, 95% CI 0.06-0.80, *P*=.02).

**Table 3:** Display of asklepians and caducei among current emblems on Internet home pages of US and Canadian medical and other health professional schools in 2014.

Schools		Longevity^a^, mean (SD, range)	Traditional asklepian, n (%)	Traditional or variant asklepian, n (%)	Traditional caduceus, n (%)	Traditional or variant caduceus, n (%)	Neither/ both, n (%)
All schools (n=482)		75.8 (58.5, 1-249)	36 (7.5)	77 (16.0)	7 (1.5)	8 (1.7)	397 (82.4)
**Medical schools**							
	All (n=159)	98.5 (61.3, 1-249)	5 (3.1)	23 (14.5)	4 (2.5)	4 (2.5)	132 (83.0)
	US^b^(n=137), PR^c^(n=4)	97.7 (62.0, 1-249)	5 (3.5)	22 (15.6)	4 (2.8)	4 (2.8)	115 (81.6)
	Canada (n=18)	104.5 (58.1, 9-185)	0 (0)	1 (6)	0 (0)	0 (0)	17 (94)
**Medical schools: region**							
	Northeast (n=44)	119.9 (72.8, 1-249)	2 (5)	8 (18)	0 (0)	0 (0)	36 (82)
	Midwest (n=33)	97.9 (53.2, 1-178)	0 (0)	5 (15)	1 (3)	1 (3)	27 (82)
	South (n=56)	87.9 (58.8, 2-207)	1 (2)	7 (13)	1 (2)	1 (2)	48 (86)
	West (n=26)	86.0 (47.0, 6-195)	2 (8)	3 (12)	2 (8)	2 (8)	21 (81)
**Other health professional schools**							
	All (n=323)	65.8 (53.5, 1-1931)	31 (9.6)	54 (16.7)	3 (0.9)	4 (1.2)	265 (82.0)
	US (n=287), PR (n=3)	65.1 (53.8, 1-193)	30 (10.3)	52 (17.9)	2 (0.7)	3 (1.0)	235 (81.0)
	Canada (n=33)	71.8 (44.0, 5-154)	1 (3)	2 (6)	1 (3)	1 (3)	30 (91)
**Other health professional schools: region**							
	Northeast (n=78)	74.3 (55.3, 1-193)	2 (3)	5 (6)	1 (1)	1 (1)	72 (92)
	Midwest (n=75)	86.9 (49.3, 1-164)	5 (7)	9 (12)	0 (0)	0 (0)	66 (88)
	South (n=106)	51.7 (46.7, 1-173)	9 (8.5)	16 (15.1)	0 (0)	0 (0)	90 (84.9)
	West (n=64)	53.9 (46.1, 1-144)	15 (23)	24 (38)	2 (3)	3 (5)	37 (58)
**Other health professional schools: osteopathic medicine**							
	All (n=40)	32.3 (33.6, 1-122)	10 (25)	14 (35)	1 (3)	1 (3)	25 (63)
	US (n=34)	33.1 (36.2, 1-122)	10 (29)	14 (41)	0 (0)	0 (0)	20 (59)
	Canada (n=6)	27.5 (11.7, 11-33)	0 (0)	0 (0)	1 (17)	1 (17)	5 (83)
**Other health professional schools: veterinary medicine**							
	All (n=35)	82.5 (45.5, 16-162)	6 (17)	10 (29)	0 (0)	0 (0)	25 (71)
	US (n=30)	82.8 (45.0, 16-162)	6 (20)	9 (30)	0 (0)	0 (0)	21 (70)
	Canada (n=5)	81.0 (58.3, 28-152)	0 (0)	1 (20)	0 (0)	0 (0)	4 (80)
**Other health professional schools: podiatric medicine**							
	All (n=10)	60.9 (47.2, 5-119)	1 (1)	4 (40)	0 (0)	0 (0)	6 (60)
	USA (n=9)	66.6 (47.1, 5-119)	1 (1)	4 (44)	0 (0)	0 (0)	5 (56)
	Canada (n=1)	10.0 (N/A^d^)	0 (0)	0 (0)	0 (0)	0 (0)	1 (100)
**Other health professional schools: dentistry**							
	All (n=74)	80.7 (47.9, 1-174)	5 (7)	7 (9)	1 (1)	1 (1)	66 (89)
	US (n=64), PR (n=1)	80.2 (49.7, 1-174)	5 (8)	7 (11)	1 (2)	1 (2)	57 (88)
	Canada (n=9)	83.8 (40.5, 43-139)	0 (0)	0 (0)	0 (0)	0 (0)	9 (100)
**Other health professional schools: pharmacy**							
	All (n=141)	64.0 (55.5, 1-193)	8 (5.7)	16 (11.3)	1 (0.7)	1 (0.7)	124 (87.9)
	US (n=130), PR (n=1)	62.2 (55.8, 1-193)	7 (5.3)	15 (11.5)	1 (0.8)	1 (0.8)	115 (87.8)
	Canada (n=10)	88.6 (49.0, 5-154)	1 (10)	1 (10)	0 (0)	0 (0)	9 (90)
**Other health professional schools: optometry**							
	All (n=23)	65.4 (46.5, 1-168)	1 (4)	3 (13)	0 (0)	1 (4)	19 (83)
	US (n=20), PR (n=1)	64.5 (47.7, 1-168)	1 (5)	3 (14)	0 (0)	1 (5)	17 (81)
	Canada (n=2)	75.5 (51.1, 47-104)	0 (0)	0 (0)	0 (0)	0 (0)	2 (100)

^a^Number of years since founding: *t* =-6.21; *P*<.001.

^b^US: the United States.

^c^PR: Puerto Rico.

^d^N/A: not applicable.

## Discussion

To our knowledge, this is the first systematic analysis of asklepian and caduceus prevalence among Internet images of medical and kindred health professional school emblems. We found that all schools’ emblems displayed the asklepian substantially more than the caduceus. This result supports the historically grounded preference for the asklepian as the symbol for healing and medicine, at least among all medical and other health professional schools, although inconsistency persists even within the constituency most expected to exemplify accurate understanding.

Human allopathic medicine in the Greco-Roman tradition appears to be the most direct descendant of Asklepios [44], yet medical schools are no more likely than health professional schools to display the traditional or any asklepian and are more likely to display the caduceus. The opportunity to improve education for correct symbol use clearly exists, as supported by the recent survey finding that only 6% of doctors knew that the asklepian is the correct symbol of medicine [14]. Medical schools and the profession can relearn and teach themselves, then promote to the public, that the asklepian represents their link to the long tradition of the healing arts and sciences.

Loss of the staff in emblems with variant asklepians may reflect not only creativity in branding, but also misunderstanding of the symbol. The staff and the serpent are a unified representation of healing through regeneration—relevant in the current era of organ, tissue, and cell transplantation. Creative use of variants can also perpetuate or increase confusion, as in the examples of the single serpent around a winged staff (Figure 2, c [30] and of the double helix conflated with the serpent symbol (Figure 2, h [35].

Fewer than 20% of current medical school and health professional school emblems in this study displayed either symbol, a finding that suggests diminished relevance of ancient symbols to the current identities of all health professional schools. Most of the schools in this study displaying neither symbol carry the crest or logo of their sponsoring universities. Health professional schools can brand their unique mission by displaying an asklepian alongside their university acronyms or insignia.

This cross-sectional study identified current symbol display, but it could not distinguish current from past symbol display. It could not identify symbol selection or change over time. For example, the Association of American Medical Colleges' emblem has changed at least twice since 1970, culminating in the current variant asklepian (Figure 2, j [37] and k [38]). Also, we could not test Friedlander’s finding that, in contrast to medical professional organizations’ preference for the asklepian, “…76% of commercial organizations were more likely to use the caduceus” [6], an observation that supports anecdotal observation of current popular and media usage. This study does propose a novel symbol classification, and its results provide increased precision in measuring asklepian versus caduceus use over time.

Evaluating global symbol use by medical and health professional schools and other health-related organizations awaits further research. Many practitioners, private and governmental [45] health care enterprises and programs, news companies, Internet knowledge providers, and others erroneously brand medicine and health care with the caduceus. Health care organizations aligning for clinically and financially accountable care in the United States and elsewhere, especially those planning to rebrand, have the opportunity to unite with all health professional schools by incorporating the asklepian, traditional or variant, into their emblems as the single symbol of a shared, interdisciplinary mission of healing.

## Appendix

Multimedia Appendix 1 Criteria for symbol definition and examples of traditional and variant asklepians and caducei.

Multimedia Appendix 2 Current and past asklepian and caduceus symbols in emblems of medical schools.

Multimedia Appendix 3 Current and past asklepian and caduceus symbols in emblems of other health professional schools.

## Abbreviations

AAMC: Association of American Medical Colleges
N/A: not applicable
OR: odds ratio
PR: Puerto Rico
US: the United States
