# Supplementary material for: Branding Asklepios and the Traditional and Variant Serpent Symbol Display Among Health Professional Schools in the United States, Puerto Rico, and Canada: A Cross-Sectional Survey
Source: JMIR Med Educ. 2016 May 25;2(1):e6. doi: 10.2196/mededu.5515 (PMC5041356; doi:10.2196/mededu.5515)
Supplement: Multimedia Appendix 1 [file mededu_v2i1e6_app1.pdf]

Multimedia Appendix 1

Asklepian: staff with one entwined serpent			Caduceus: double-winged wand with two symmetrically entwined serpents		Display of both Asklepian and Caduceus
Traditional	Variant 1	Variant 2	Traditional	Variant	
Knobby or branching, rough or wooden-appearing staff with notched or asymmetric top, and one serpent	Smooth rod with or without a solid or hollow knob at the top, and one serpent	Double-winged or flame-topped, smooth staff and one serpent; or serpent only	Double-winged wand and two serpents	Double-winged wand or two serpents	

Note: For all asklepians and caducei, the definitions accept either serpent chirality (facing left or right) and any number of serpent coils around the staff or wand.

Examples of Traditional and Variant Asklepians and Caducei

Medical Schools (see key below, p. 8)

<u>Asklepian</u>			<u>Caduceus</u>		<u>Both</u>
Traditional	Variant 1	Variant 2	Traditional	Variant	Asklepian and Caduceus
1	4	7	10	12	15

2

5

8

9

6

3

11

13

14

15

Other Health Profession Schools (see key below, p. 8) *Osteopathic Medicine*

Asklepian			Caduceus		Both
Traditional	Variant 1	Variant 2	Traditional	Variant 1	
16<br>17	1<br>8	none	none	none	none

Other Health Profession Schools *Veterinary Medicine*

Asklepian			Caduceus		Both
Traditional	Variant 1	Variant 2	Traditional	Variant 1	
19<br>20	21<br>22	23	none	24	none

Other Health Profession Schools *Podiatric Medicine*

Asklepian			Caduceus		Both
Traditional	Variant 1	Variant 2	Traditional	Variant 1	
25	27	28	none	none	none

26	29	none	none	none

Other Health Profession Schools *Dentistry*

Asklepian			Caduceus		Both
Traditional	Variant 1	Variant 2	Traditional	Variant	
Instrument (cautery) or traditional staff and one serpent, and all 4 components: overlying O, *omega*, and Δ, *delta*, with leaves and berries	Instrument (cautery) or traditional staff and one serpent, and 3 of 4 components	Instrument (cautery) or traditional staff and one serpent, and and 2 or less of 4 components	Instrument (cautery) or traditional staff and double wings and two serpents, and all 4 components; or, traditional wand and double wings and two serpents	Instrument (cautery) or traditional staff and two wings and 2 serpents, and ≤ 3 of 4 components	
30	31	33	34	35	none
	32				

Other Health Profession Schools *Pharmacy*

Asklepian			Caduceus		Both
Traditional	Variant 1	Variant 2	Traditional	Variant	
Chalice with both bowl and stem, OR both mortar and pestle, AND one serpent	Chalice bowl without stem, OR mortar or pestle, AND one serpent	Chalice bowl without stem, OR mortar and pestle, OR one serpent	Chalice with both bowl and stem, OR both mortar and pestle, AND two serpents	Chalice bowl without stem, OR mortar or pestle, OR two serpents	
3	39	41	43	4	45
6	40	42		4	
37					
38					

Other Health Profession Schools *Optometry*

Asklepian			Caduceus		Both
Traditional	Variant 1	Variant 2	Traditional	Variant 1	
46	47	48<br>49	none	50	none

Key (hyperlinks) for Examples of Traditional and Variant Asklepians and Caducei

1 Case Western Reserve University School of Medicine

2 Stanford University School of Medicine

3 Queen's University Faculty of Health Sciences

4 Geisel School of Medicine at Dartmouth

5 Southern Illinois University School of Medicine

6 University of Wisconsin School of Medicine and Public Health

7 New York Medical College

8 Memorial University of Newfoundland Faculty of Medicine

9 Association of American Medical Colleges (2015)

10 Louisiana State University School of Medicine in New Orleans

11 Sanford School of Medicine The University of South Dakota

12 University of Illinois College of Medicine

13 University of Hawaii, John A. Burns School of Medicine

14 Association of American Medical Colleges (2006)

15 Morehouse School of Medicine

16 Lincoln Memorial University-DeBusk College of Osteopathic Medicine

17 Pacific Northwest University of Health Sciences College of Osteopathic Medicine

18 Campbell University School of Osteopathic Medicine

19 Auburn University College of Veterinary Medicine

20 Iowa State University College of Veterinary Medicine

21 Kansas State University College of Veterinary Medicine

22 Association of American Veterinary Medical Colleges

23 Michigan State University College of Veterinary Medicine

24 Colorado State University College of Veterinary Medicine

25 Des Moines University College of Podiatric Medicine and Surgery

26 Midwestern Arizona School of Podiatric Medicine

27 California School of Podiatric Medicine at Samuel Merritt University

28 Barry University School of Podiatric Medicine

29 Western University of Health Sciences College of Podiatric Medicine

30 Herman Ostrow School of Dentistry of University of Southern California

31 Indiana University School of Dentistry

32 Southern Illinois University School of Dental Medicine

33 University of Detroit Mercy School of Dentistry

34 University of Utah School of Dentistry

35 University of Manitoba Faculty of Dentistry

36 Chicago State University College of Pharmacy

37 Campbell University College of Pharmacy and Health Sciences

38 Canadian Pharmacists Association/Association des pharmaciens du Canada

39 Ohio Northern University College of Pharmacy

40 University of Pittsburgh School of Pharmacy

41 California Northstate University College of Pharmacy

42 University of Cincinnati James L. Winkle College of Pharmacy

43 University of Hawaii at Hilo Daniel K. Inouye College of Pharmacy

44 Drake University College of Pharmacy and Health Sciences

45 University of Manitoba Faculty of Pharmacy

46 Midwestern University Arizona College of Optometry

47 Nova Southeastern University College of Optometry

48 Illinois College of Optometry

49 Western University of Health Sciences College of Optometry

50 Inter American University of Puerto Rico School of Optometry
