# Supplementary material for: Branding Asklepios and the Traditional and Variant Serpent Symbol Display Among Health Professional Schools in the United States, Puerto Rico, and Canada: A Cross-Sectional Survey
Source: JMIR Med Educ. 2016 May 25;2(1):e6. doi: 10.2196/mededu.5515 (PMC5041356; doi:10.2196/mededu.5515)
Supplement: Multimedia Appendix 2 [file mededu_v2i1e6_app2.pdf]

Multimedia Appendix 2

Key

Bold: School displays traditional symbol; variants are denoted as [1] & [2], as defined in Section 1; [b] denotes school displaying both asklepian and caduceus.

• Schools (22) Displaying Asklepians in Emblems (24) on Current Homepages			
Geisel School of Medicine at Dartmouth[1]		Hofstra North Shore - LIJ School of Medicine[1]	
Loma Linda University School of Medicine[1]		Medical College of Wisconsin[1]	
**Mercer University School of Medicine**		New York Medical College[2]	
Morehouse School of Medicine[2b]		Northern Ontario School of Medicine[1]	
Ponce School of Medicine and Health Sciences[1]	x	Note: Switched to caduceus between 12/2013[x] and 12/2014[y]	y

Rutgers, Robert Wood Johnson Medical School[2]		San Juan Bautista School of Medicine[1]	
Southern Illinois University School of Medicine[1]		**Stanford University School of Medicine**	
Uniformed Services University of the Health Sciences F. Edward Hebert School of Medicine[2]		Uniformed Services University of the Health Sciences F. Edward Hebert School of Medicine[2]	
Universidad Central del Caribe School of Medicine[2]		**University of Nevada School of Medicine**	
University of Puerto Rico School of Medicine[2]		**University of Rochester School of Medicine and Dentistry**	
Wayne State University School of Medicine[1]		Western Michigan University School of Medicine[1]	
Wright State University Boonshoft School of Medicine[1]		**Yale University School of Medicine**	

- Additional Schools (48) Displaying Asklepians in Emblems among Current or Past Images (50)

Albany Medical College[2]	in-Training the agora of the medical student community		
Baylor College of Medicine[2]		Boston University School of Medicine[1]	
**Case Western Reserve University School of Medicine**		**Columbia University College of Physicians and Surgeons**	
**Duke University School of Medicine**		**Duke University School of Medicine**	
**Emory University School of Medicine**		Florida State University College of Medicine[2]	
Howard University College of Medicine[1]		McGill University Faculty of Medicine[1]	

**Meharry Medical College**[2,b]		Memorial University of Newfoundland Faculty of Medicine[2]	
New York University School of Medicine[2]		Northeast Ohio Medical University[1]	
**Oakland University William Beaumont School of Medicine**		**Queen's University Faculty of Health Sciences**	
**State University of New York Upstate Medical University**		Temple University School of Medicine[2]	
**Université de Laval Faculté de Médicine**		University of Alabama School of Medicine[2]	
University of Arizona College of Medicine-Phoenix[1]			
University of Alberta Faculty of Medicine and Dentistry[1]			

University of Arkansas for Medical Sciences College of Medicine[1]		**University of British Columbia Faculty of Medicine**	
University of California, Davis, School of Medicine[1]		University of California, Irvine, School of Medicine[2,b]	
University of Colorado School of Medicine[1]		University of Manitoba Faculty of Medicine[2]	
University of Illinois College of Medicine[2,b]		University of Illinois College of Medicine	
University of Massachusetts Medical School[2]		University of Miami Leonard M. Miller School of Medicine[1]	
**University of Michigan Medical School**		**University of Minnesota Medical School**	
University of Mississippi School of Medicine[1]			
University of New Mexico School of Medicine[1]			

University of North Dakota School of Medicine and Health Sciences[1]		**University of Oklahoma College of Medicine**<br><br>Link not found 12/19/14	
University of Ottawa Faculty of Medicine[2]		University of Saskatchewan College of Medicine[1]	
**University of South Alabama College of Medicine**		**University of South Florida Morsani College of Medicine**	
**University of Tennessee Health Science Center College of Medicine**		University of Texas Medical School at Houston[2]	
University of Toronto Faculty of Medicine[1]		University of Vermont College of Medicine[1]	
University of Wisconsin School of Medicine and Public Health[2]		**West Virginia University School of Medicine**	
• Schools (4) Displaying Caducei in Emblems (4) on Current Homepages			

**East Tennessee State University James H. Quillen College of Medicine**		University of Hawaii, John A. Burns School of Medicine[1]	
**University of Kentucky College of Medicine**			
**University of Utah School of Medicine**			
• Additional Schools (14) Displaying Caducei in Emblems among Current or Past Images (14)			
**George Washington University School of Medicine and Health Sciences**		**Harvard Medical School**<br>Division of Medical Ethics link not found 12/19/14	
**Icahn School of Medicine at Mount Sinai**		**Louisiana State University School of Medicine**<br>**in New Orleans**	
**Marshall University Joan C. Edwards**<br>**School of Medicine**		**Meharry Medical College**[b]	

**Morehouse School of Medicine**[b]	MSMNAA MOREHOUSE SCHOOL OF MEDICINE NATIONAL ALUMNI ASSOCIATION		
University of California, Irvine, School of Medicine[1,b]	Students for Integrative Medicine University of California, Irvine		
University of Cincinnati College of Medicine[1]	UNIVERSITY OF CINCINNATI 1819	University of Illinois College of Medicine[1,b]	
**University of Louisville School of Medicine**	LOUISVILLE MEDICINE	**University of Medicine and Dentistry of New Jersey- New Jersey Medical School**	UNIVERSITY OF MEDICINE & DENTISTRY OF NEW JERSEY UMDNJ
**University of North Carolina at Chapel Hill School of Medicine**	Pediatric Rheumatology Casebook	**Sanford School of Medicine The University of South Dakota**	USD
