# Supplementary material for: Branding Asklepios and the Traditional and Variant Serpent Symbol Display Among Health Professional Schools in the United States, Puerto Rico, and Canada: A Cross-Sectional Survey
Source: JMIR Med Educ. 2016 May 25;2(1):e6. doi: 10.2196/mededu.5515 (PMC5041356; doi:10.2196/mededu.5515)
Supplement: Multimedia Appendix 3 [file mededu_v2i1e6_app3.pdf]

Multimedia Appendix 3

Key

Bold: School displays traditional symbol; variants are denoted as [1] & [2], as defined in Section 1; [b] denotes school displaying both asklepian and caduceus.

a. *Osteopathic Medicine*

- Schools (14) Displaying Asklepians in Emblems (14) on Current Homepages

Alabama College of Osteopathic Medicine[1]	**Arizona College of Osteopathic Medicine, Midwestern University**
**Chicago College of Osteopathic Medicine, Midwestern University**	**Des Moines University College of Osteopathic Medicine**
Edward Via College of Osteopathic Medicine - Carolinas Campus[1]	Edward Via College of Osteopathic Medicine - Virginia Campus[1]
Liberty University College of Osteopathic Medicine[1]	**Lincoln Memorial University-DeBusk College of Osteopathic Medicine**
Ohio University Heritage College of Osteopathic Medicine[1]	**Pacific Northwest University of Health Sciences College of Osteopathic Medicine**
**Rocky Vista University College of Osteopathic Medicine**	**Touro College of Osteopathic Medicine - New York**
**West Virginia School of Osteopathic Medicine**	**Western University of Health Sciences College of Osteopathic Medicine of the Pacific**

- Additional Schools (10) Displaying Asklepians in Emblems (10) among Current or Past Images

Campbell University School of Osteopathic Medicine[1]	**Lake Erie College of Osteopathic Medicine** **Lake Erie College of Osteopathic Medicine Bradenton**
Michigan State University College of Osteopathic Medicine[1]	**New York Institute of Technology College of Osteopathic Medicine**
**Nova Southeastern University**	**Touro University Nevada College of Osteopathic**
